# Complete mitochondrial genome of the southern painted turtle (*Chrysemys dorsalis*, Testudines: Emydidae) in Korea

**DOI:** 10.1080/23802359.2023.2301025

**Published:** 2024-01-08

**Authors:** Ye-Eun Ji, Kyoung-Hee Park, Jae-Hyeok Choi, Jaehong Park, Ha-Cheol Sung, Dong-Hyun Lee

**Affiliations:** a School of Biological Sciences and Biotechnology Graduate School, Chonnam National University, Gwangju, Korea; b Research Center of Ecomimetics, Chonnam National University, Gwangju, Korea; c Department of Biological Sciences, College of Natural Sciences, Chonnam National University, Gwangju, Korea

**Keywords:** *Chrysemys dorsalis*, Emydidae, Mitochondrial genome

## Abstract

The complete mitochondrial genome of *Chrysemys dorsalis* in Korea was sequenced and characterized. The mitochondrial genome is 17,258 bp in length and the GC content is 39%. It is constituted of 37 genes, 13 protein-coding genes, 22 transfer RNA genes, 2 ribosomal RNA genes, and a noncoding region. Phylogenetic analysis reveals that *C. dorsalis* forms a monophyletic group with *C. picta* turtles but is distinctly separated from them, aligning with previous findings. In Korea, *C. dorsalis* forms a discrete clade, separate from both native and invasive turtle species. No evidence of genetic disturbance or intermingling is observed. This is the first case of a complete mitochondrial genome from *C. dorsalis* and provides crucial data for understanding *C. dorsalis* and managing invasive species effectively, emphasizing the need for continued mitochondrial genome data accumulation.

## Introduction

*Chrysemys dorsalis* (Agassiz 1857), commonly known as the southern painted turtle, belongs to the Emydidae family and is recognizable by a prominent red stripe running down the center of its carapace. Initially considered a subspecies of *C. picta*, subsequent studies argued for the recognition of *C. dorsalis* as a distinct species based on mitochondrial DNA sequences (Starkey et al. 2003). This conclusion was supported by the following study and accepted by other societies (Jensen et al. 2015).

The natural habitat of *C. dorsalis* extends across the south-central United States, including regions from southern Illinois and Missouri to the Gulf coast of Louisiana and parts of Alabama (McAllister et al. 2007). In addition to the United States, *C. dorsalis* has also been found in other countries, including Korea (GBIF Secretariat 2022).

Most of the invasive species found in the wild result from release by humans (Oh and Hong 2007; Mun et al. 2013). This introduction causes serious damage to native ecosystems and threatens biodiversity (Nentwig et al. 2018). In Korea, the number of invasive species has been steadily increasing, particularly due to the pet trade (Koo et al. 2020).

Despite the importance of managing the invasive species *C. dorsalis* in Korea, there is few information available about this turtle, and its complete mitochondrial genome remains uncharacterized. This study aims to present the complete mitochondrial genome sequence of C. dorsalis and investigate its phylogenetic relationship with native and invasive turtle species in Korea.

## Materials and methods

A specimen of *C. dorsalis* was collected from Gwangju, Korea (35°6'14.66"N, 126°54'17.29"E), and the total genomic DNA was extracted from the tail using the DNeasy Blood & Tissue kit (Qiagen, Valencia, CA) following the manufacturer’s instructions. The extracted DNA sample was deposited at the Museum of Wildlife, located in Research Center of Ecomimetics, Chonnam National University, Korea (https://biology.jnu.ac.kr; Ha-Cheol Sung; shcol2002@jnu.ac.kr) under Specimen voucher number: 2023-RCE-CD001. Mitochondrial genome analysis was performed using Illumina NovaSeq X plus platform (Illumina, San Diego, CA) by Macrogen (Seoul, Korea). Raw sequence data were assessed using FastQC, and adaptor trimming and quality filtering were performed using Trimmomatic (Andrews 2010; Bolger et al. 2014). Subsequently, *de novo* assembly was conducted using SPAdes 3.15.0, and the filtered reads were aligned using BLAST (Altschul et al. 1990; Bankevich et al. 2012). Finally, the complete sequence was annotated using MITOS2 web server (Bernt et al. 2013).

To investigate the phylogenetic position of *C. dorsalis*, the complete mitochondrial genome sequences of 16 species in *Testudines* were obtained from GenBank, and the phylogenetic tree was constructed using MEGA X software (Kumar et al. 2018). The sequences were aligned using MUSCLE algorithm, and the phylogenetic tree was generated using maximum likelihood method with the GTR + G model and 1000 bootstrap replicates (Waddell and Steel 1997; Edgar 2004). The GTR + G substitution model was selected as the best-fit model by MEGA X.

## Results

**Figure 1.**
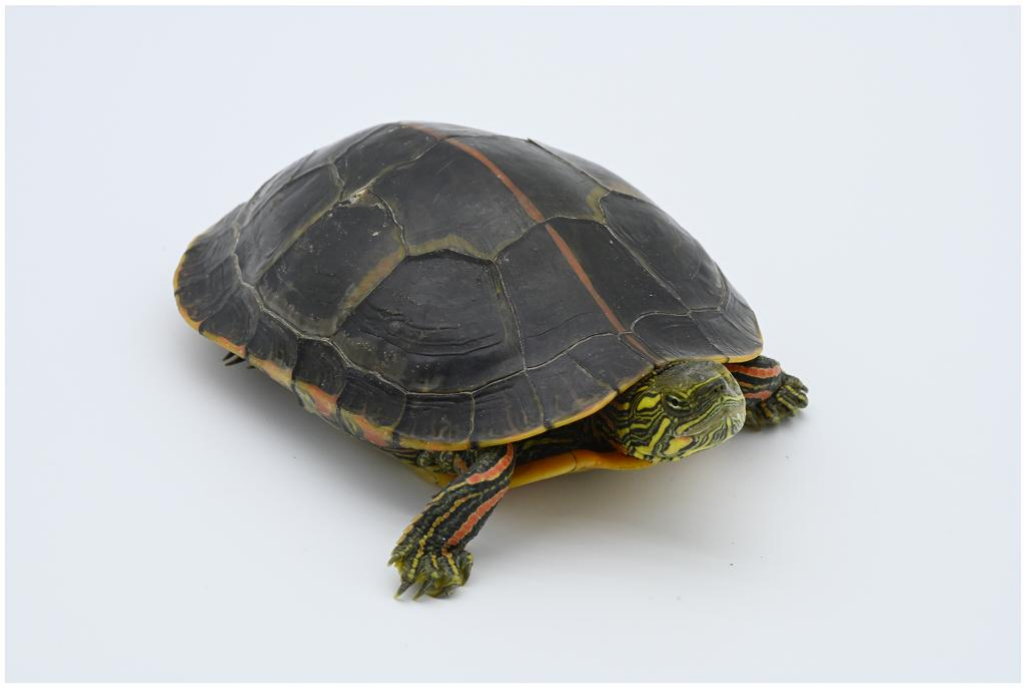
Mitochondrial genome map of *Chrysemys dorsalis*. Genes encoded on heavy strand are written outside the circle and genes on light chain inside the circle.

The complete mitochondrial genome of *C. dorsalis* is 17,258 bp in length and has been deposited in GenBank (Accession number: OR253894). It comprises 13 protein-coding genes (PCGs), 22 transfer RNA (tRNA) genes, 2 ribosomal RNA (rRNA) genes, and a putative long non-coding control region. Among these, 12 PCGs, 14 tRNA genes, and 2 rRNA genes are encoded in the heavy strand, while 1 PCG (NADH dehydrogenase subunit 6) and 8 tRNA genes are encoded in the light strand (Figure 1). Of the 13 PCGs, 11 have canonical mitochondria start codons, including AUG for *COX2*, *ATP8*, *ATP6*, *COX3*, *ND4L*, *ND5*, *ND6*, and *Cytb*, and AUA for *ND1*, *ND2*, and *ND3. COX1* and *ND4* have alternative initiation codon (GUG). Additionally, *COX3* and *ND4* have incomplete stop codon ending with UA and U, respectively.

The nucleotide composition of the *C. dorsalis* mitochondrial genome is as follows: *A* = 34.4%, *T* = 26.6%, *G* = 12.9%, and *C* = 26.1%. It closely resembles the nucleotide composition of *C. p. bellii* from China (KF874616; *A* = 34.4%, *T* = 26.6%, *G* = 13.0%, and *C* = 26.0%) and *C. picta* from the USA (AF069423; *A* = 34.4%, *T* = 26.8%, *G* = 12.8%, and *C* = 25.9%). The sequence of *C. dorsalis* from Korea shows a high similarity of 98% with *C. p. bellii* from China and *C. picta* from USA, it is less similar to *Pseudemys peninsularis* from Korea (OM935748; 93%), *P. concinna* from Korea (OM935747; 93%), *Trachemys scripta elegans* from Korea (MW019443; 90%), *Mauremys reevesii* from Korea (FJ469674; 81%), or *Pelodiscus sinensis* from Korea (AY962573; 76%).

**Figure 2.**
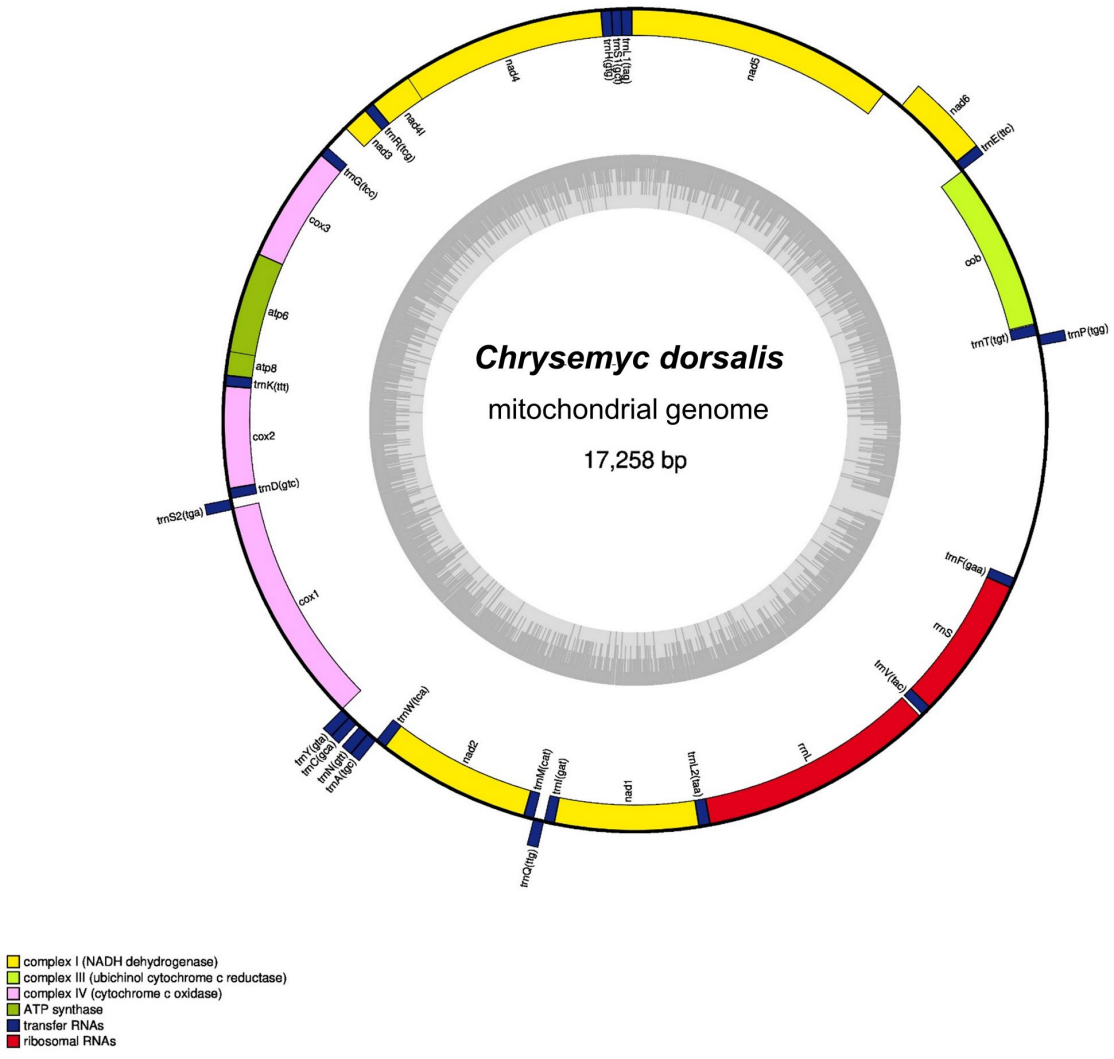
Phylogenetic tree of *C. dorsalis* and other related species based on mitochondrial genome sequences. Phylogenetic analysis was performed using MEGA X software. GenBank accession numbers of each mt genome sequence are given in the bracket after the species name, and the following sequences were used: AF069423 (Mindell et al. 1999), KF874616 (Jiang et al. 2016), OM935748 (Chung et al. 2022), OM935747 (Park et al. 2022), KM216748 (Yu et al. 2016), MW019443 (Park et al. 2021), FJ392294 (Russell and Beckenbach, 2008), KC333650 (Fang et al. 2013), FJ871126 (reference unavailable), FJ469674 (Shin et al. 2015), KJ700438 (reference unavailable), AP019398 (Asami et al. 2019), AY962573 (Jung et al. 2006), MG431983 (Zhang et al. 2019), AY687385 (reference unavailable), and AF039066 (Zardoya and Meyer, 1998). The bootstrap value based on 1,000 replicates is represented on each node. *Pelomedusa subrufa* was used as outgroup to root the tree.

To explore the mitogenomic relationship between *C. dorsalis* and both native and invasive species in Korea, we retrieved complete mitochondrial genome sequences of the respective species from GenBank. Subsequently, a phylogenetic tree was constructed. The analysis revealed that *C. dorsalis* forms a monophyletic group with other *C. picta* turtles, but is clearly separated from them with a high bootstrap score. Moreover, *C. dorsalis* in Korea is not clustered with other turtles found in Korea, regardless of whether they are native species (*M. reevesii* and *P. sinensis*) or the invasive species (*P. peninsularis*, *P. concinna*, and *T. s. elegans*) (Figure 2).

**Figure 3.**
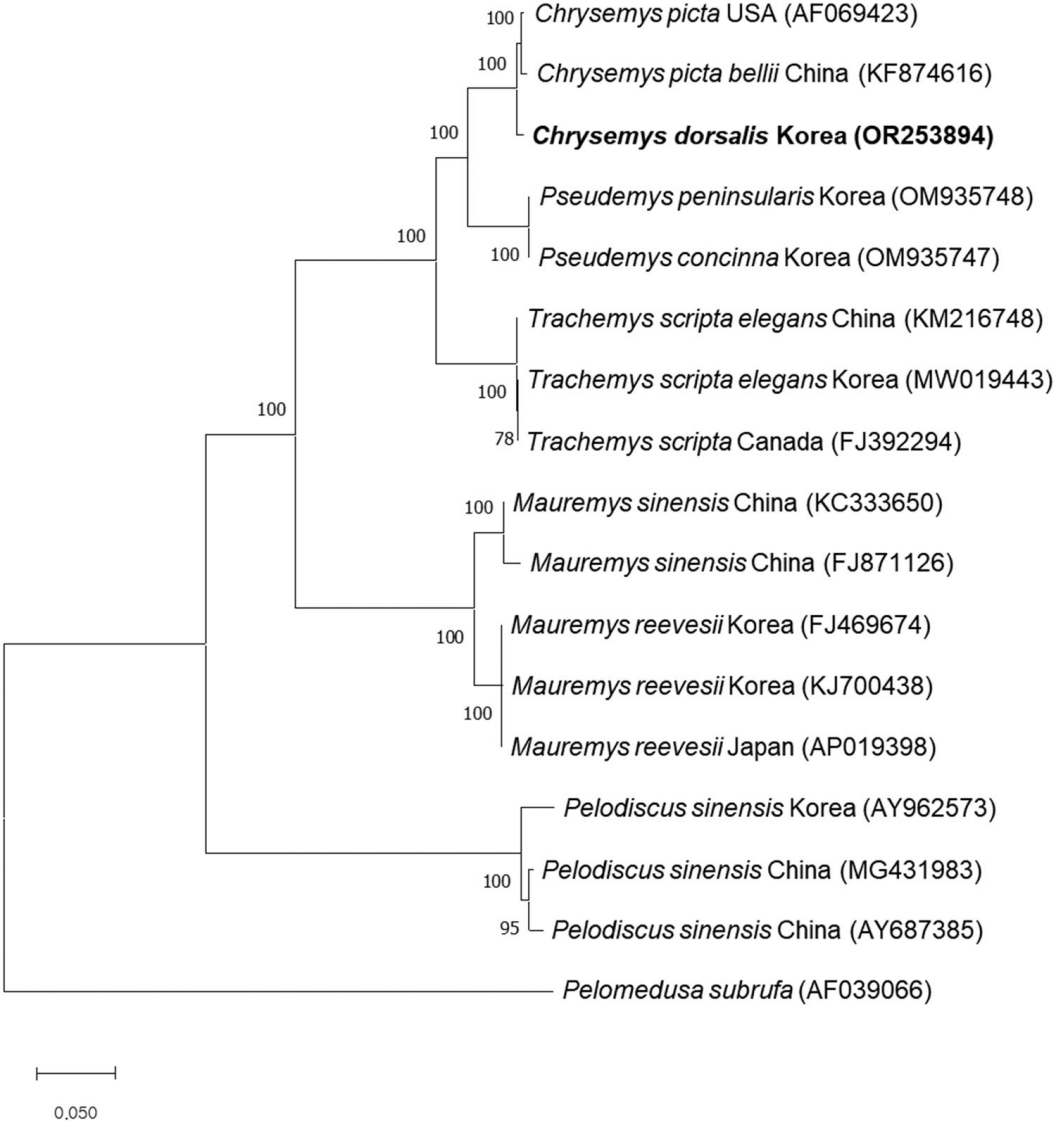
Reference image for *Chrysemys dorsalis*. This picture was taken by the authors..

## Discussion and conclusion

We sequenced the complete mitochondrial genome of *C. dorsalis* and revealed the phylogenetic relationship with other turtles by constructing a phylogenetic tree. In the phylogenetic tree, *C. dorsalis* makes a monophyletic group with the other *C. picta* turtles, but they are separated with a high bootstrap score, in line with findings from a prior study (Starkey et al. 2003). In contrast, *C. dorsalis* in Korea forms a distinct clade when compared to other turtles, including both the native species (*M. reevesii* and *P. sinensis*) and the invasive species (*P. peninsularis*, *P. concinna*, and *T. s. elegans*), aligning with results from other research resources (Fritz et al. 2011). There is currently no evidence of genetic disturbance or intermingling between invasive species and either other invasive species or native species. However, a comprehensive investigation into the impact of *C. dorsalis* on native species and their habitat requires the accumulation of mitochondrial genome data over an extended period. This study will provide previously unavailable information and offer new insights and crucial datas for understanding *C. dorsalis* and managing the invasive species including *C. dorsalis* effectively.

## Supplementary Material

Supplemental Material

## Data Availability

GenBank accession number from the complete mitochondrial genome of *Chrysemys dorsalis* (OR253894) has been registered with the NCBI database (https://www.ncbi.nlm.nih.gov/OR253894). The associated BioProject, BioSample, and SRA accession numbers are PRJNA993826, SAMN36409324, and SRR25242851, respectively.
