# Supplementary material for: Complete mitochondrial genome of the southern painted turtle (Chrysemys dorsalis, Testudines: Emydidae) in Korea
Source: Mitochondrial DNA B Resour. 2024 Jan 8;9(1):70–4. doi: 10.1080/23802359.2023.2301025 (PMC10786420; doi:10.1080/23802359.2023.2301025)
Supplement: Supplemental Material [file TMDN_A_2301025_SM2783.docx]

**Supplementary materials**

**Complete mitochondrial genome of the southern painted turtle (*Chrysemys dorsalis*, Testudines: Emydidae) in Korea.**

Ye-Eun Ji^a^*, Kyung-Hee Park^a^*, Jae-Hyuk Choi^a^, Jaehong Park^a^, Ha-Cheol Sung^b,c^, Dong-Hyun Lee^b,c^

^a^School of Biological Sciences and Biotechnology Graduate School, Chonnam National University, Gwangju, Korea; ^b^Research Center of Ecomimetics, Chonnam National University, Gwangju, Korea; ^c^Department of Biological Sciences, College of Natural Sciences, Chonnam National University, Gwangju, Korea

*These authors contributed equally to this work.

**CONTACT**

Ha-Cheol Sung (shcol2002@jnu.ac.kr); Dong-Hyun Lee (donghyunlee73@jnu.ac.kr); Department of Biological Sciences, College of Natural Sciences, Chonnam National University, 77, Yongbong-ro, Buk-gu, Gwangju, 61186, Korea


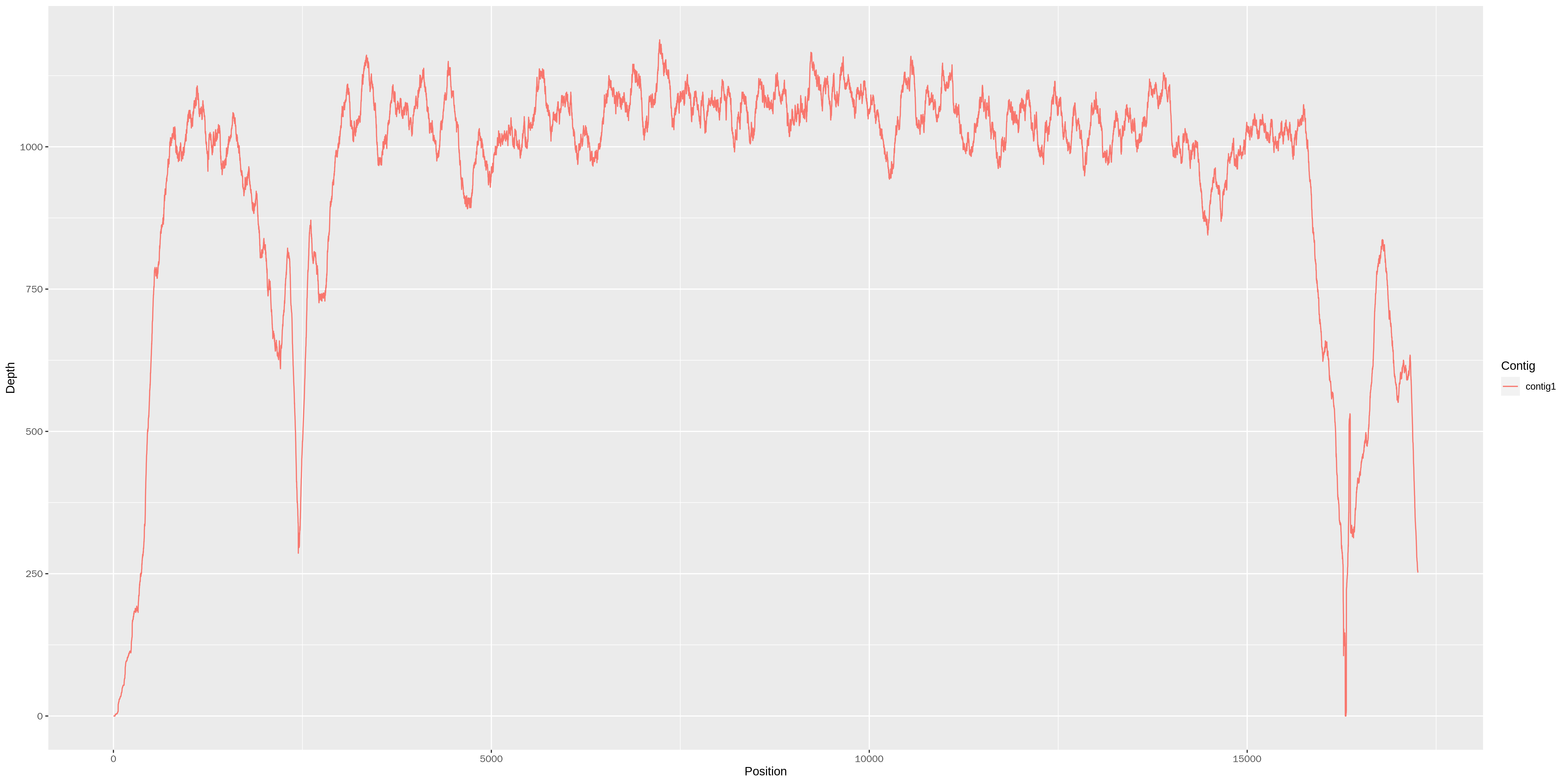


**Supplementary Figure 1.** Coverage depth map of *Chrysemys dorsalis*.
